# Infiltration of M1, but not M2, macrophages is impaired after unilateral ureter obstruction in *Nrf2*-deficient mice

**DOI:** 10.1038/s41598-017-08054-2

**Published:** 2017-08-18

**Authors:** Yuji Sogawa, Hajime Nagasu, Shigeki Iwase, Chieko Ihoriya, Seiji Itano, Atsushi Uchida, Kengo Kidokoro, Shun’ichiro Taniguchi, Masafumi Takahashi, Minoru Satoh, Tamaki Sasaki, Takafumi Suzuki, Masayuki Yamamoto, Tiffany Horng, Naoki Kashihara

**Affiliations:** 1 Department of Nephrology and Hypertension, Kawasaki Medical School, Kurashiki, Okayama Japan; 2 Department of Human Genetics, University of Michigan, Ann Arbor, Michigan USA; 3 Department of Molecular Oncology, Shinshu University Graduate School of Medicine, Matsumoto, Nagano Japan; 4 Division of Inflammation Research, Center for Molecular Medicine, Jichi Medical University, Shimotsuke, Tochigi Japan; 5 Department of Medical Biochemistry, Tohoku University Graduate School of Medicine, Sendai, Miyagi Japan; 6 Department of Genetics & Complex Diseases, Harvard T.H. Chan School of Public Health, Boston, Massachusetts USA

## Abstract

Chronic inflammation can be a major driver of the failure of a variety of organs, including chronic kidney disease (CKD). The NLR family pyrin domain-containing 3 (NLRP3) inflammasome has been shown to play a pivotal role in inflammation in a mouse kidney disease model. Nuclear factor erythroid 2-related factor 2 (Nrf2), the master transcription factor for anti-oxidant responses, has also been implicated in inflammasome activation under physiological conditions. However, the mechanism underlying inflammasome activation in CKD remains elusive. Here, we show that the loss of Nrf2 suppresses fibrosis and inflammation in a unilateral ureter obstruction (UUO) model of CKD in mice. We consistently observed decreased expression of inflammation-related genes *NLRP3* and *IL-1β* in *Nrf2*-deficient kidneys after UUO. Increased infiltration of M1, but not M2, macrophages appears to mediate the suppression of UUO-induced CKD symptoms. Furthermore, we found that activation of the NLRP3 inflammasome is attenuated in *Nrf2*-deficient bone marrow–derived macrophages. These results demonstrate that Nrf2-related inflammasome activation can promote CKD symptoms via infiltration of M1 macrophages. Thus, we have identified the Nrf2 pathway as a promising therapeutic target for CKD.

## Introduction

Nuclear factor erythroid 2-related factor 2 (Nrf2) is a master transcription factor for anti-oxidant and detoxification responses^1, 2^. Cellular Nrf2 levels are precisely regulated by transcriptional and post-transcriptional mechanisms to ensure adequate responses to oxidative stresses. Under physiological conditions, adaptor protein kelch-like ECH-associated protein 1 (Keap1) binds to the Nrf2 protein and accelerates proteasomal degradation of Nrf2. With oxidative stress, Keap1 releases Nrf2, which allows Nrf2 to translocate into the nucleus to activate the expression of anti-oxidant-related genes, such as *NAD(P)H quinone oxidoreductase* (*NQO1*), *heme oxygenase 1* (*HO-1*), and *glutamate-cysteine ligases* (*GCLM* and *GCLC*)^3^. These anti-oxidant genes, especially *HO-1*, are protective against kidney dysfunction, including chronic kidney disease (CKD) and acute kidney injury^4–6^.

Nrf2 has been reported to play a role in a variety of pathologic kidney conditions. However, recent reports have also demonstrated that Nrf2 is required for activation of the nucleotide-binding domain, leucine-rich repeat containing protein (NLRP3) inflammasome^7^.

Inflammasomes are multimeric protein complexes that form in the cytosol in response to either exogenous pathogens or endogenous danger signals and induce proinflammatory effects. Although inflammasomes are an innate immune response to eliminate infectious pathogens from tissues, chronic inflammation can be a major driver of the failure of a variety of organs, including the development of CKD. Activation of inflammasomes is a key mediator in chronic inflammation, but aberrant inflammasome activation is involved in many different diseases, including atherosclerosis and heart failure^8–10^. NLRP3, a Nod-like receptor family member, and an adaptor apoptosis-associated speck-like protein with a caspase recruitment domain (ASC) within inflammasomes, activate caspase-1, which in turn promotes maturation of the proinflammatory cytokines interleukin-1 beta (IL-1β) and IL-18^11, 12^. Recently, the NLRP3 inflammasome has been shown to play a pivotal role in inflammation in murine kidney disease models^13–16^. Of note, *Nrf2* knockout has led to the amelioration of several metabolic disorders and cardiovascular diseases, including atherosclerosis^17–21^, obesity^22^, and type 2 diabetes^23^, all of which are typically associated with chronic inflammation. These observations raise the intriguing possibility that Nrf2 may affect kidney diseases via inflammasome activation in addition to, or in lieu of, its classic role as an oxidative stress response factor. However, the role of Nrf2-related inflammasome activation in kidney disease is still unclear.

To better understand the role of Nrf2 in the progression of kidney diseases, we generated a unilateral ureter-ligated mouse model in wild-type (WT) and *Nrf2*-knockout (KO) backgrounds. We showed that Nrf2-dependent inflammasome activation was important for maintaining inflammatory macrophages, such as M1 macrophages, but not M2 macrophages. We also found that caspase-1 inhibitors were able to suppress M1 macrophage infiltration. These data increase our understanding of the role of Nrf2 in kidney diseases.

## Results

### Nrf2 deficiency suppresses inflammation and fibrosis in a UUO model

**Figure 1:**
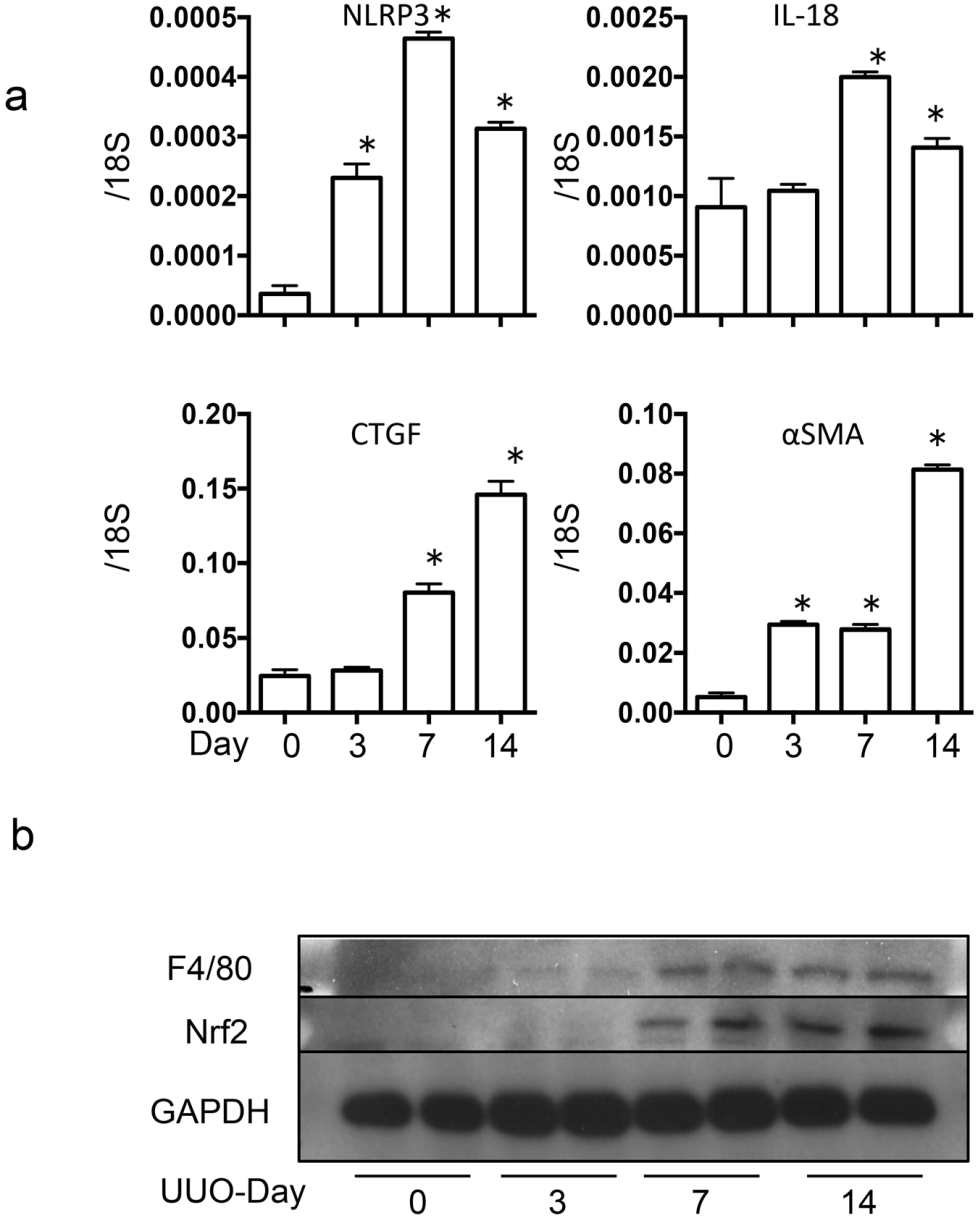
Trends of inflammatory and fibrogenic gene expression in unilateral ureter obstruction. These data were obtained from whole kidney tissue from 5 mice in each group. (**a**) Expression of inflammatory genes (*nucleotide-binding domain, leucine-rich repeat containing protein* [*NLRP3*] and *interleukin-18* [*IL-18*]) and fibrogenic genes (*connective tissue growth factor* [*CTGF*] and *alpha smooth muscle actin* [*α-SMA*]) were assessed with quantitative reverse transcription PCR (qRT-PCR). The mRNA expression levels of each gene were normalized to the expression of 18S. *p < 0.05 relative to Day 0. (**b**) Representative western blotting experiments and analysis data for F4/80, nuclear factor erythroid 2-related factor 2 (Nrf2) and GAPDH.

**Figure 2:**
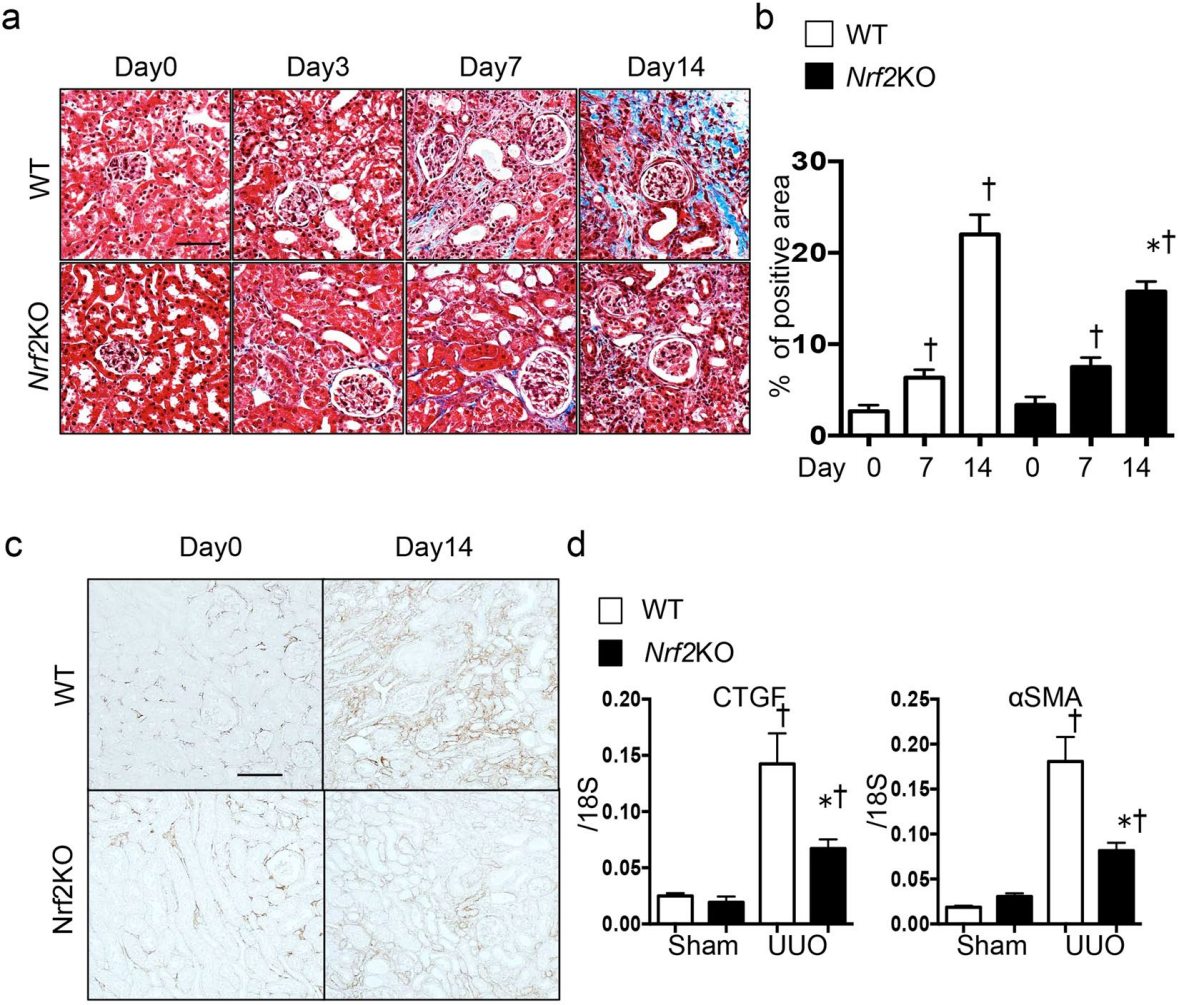
Fibrosis in wild-type (WT) and *nuclear factor erythroid 2-related factor 2*-knockout (*Nrf2*-KO) mice after unilateral ureter obstruction (UUO). These data were obtained from whole kidney tissues from 10 or more mice in each group. (**a**) Fibrosis was assessed with Masson’s trichrome staining. Representative images are shown. (**b**) The tubulointerstitial fibrosis area (%) was evaluated as the blue-stained positive area in the Masson’s trichrome staining. Scale bar = 50 μm. *p < 0.05 relative to WT-UUO on the same day after UUO. ^†^p < 0.05 relative to Sham. (**c**) Collagen IV staining was performed to evaluate interstitial fibrosis. Representative images are shown. Scale bar = 100 μm. (**d**) The fibrosis-associated genes (*connective tissue growth factor* [*CTGF*] and *alpha smooth muscle actin* [*α-SMA*]) were assessed with quantitative reverse transcription PCR (qRT-PCR). *p < 0.05 relative to WT-UUO. ^†^p < 0.05 relative to Sham.

The murine unilateral ureter obstruction (UUO) model is one of the most useful models of kidney inflammation and fibrosis. First, we examined the kinetics of the mRNA levels of inflammasome-related genes and fibrosis genes during UUO-induced kidney injury. Although the inflammasome-related genes *NLRP3* and *IL-18* peaked 7 days after UUO, the induction of fibrosis genes was delayed; 14 days after UUO, the fibrosis genes remained upregulated (Fig. 1a). These results are consistent with a model in which inflammation is instructive for kidney fibrosis. Interestingly, the increased levels of the macrophage marker F4/80 coincided with the upregulation of Nrf2 during this time, which suggests that Nrf2 may function through macrophages in UUO-induced inflammation (Fig. 1b). To test the role of Nrf2, we induced UUO in WT and *Nrf2*-KO mice, in which the *Nrf2* exon 4 was deleted^24^. We then performed a series of histological assessments after the UUO procedure. The extent of fibrosis was successively evaluated with Masson’s trichrome staining (Days 0, 3, 7, and 14). Although the WT and *Nrf2*-KO mice showed similar UUO-induced fibrosis by Day 7, we noted a statistically significant decrease in fibrosis, measured by fibrosis area, in the *Nrf2-*KO kidneys compared with the WT kidneys (Fig. 2a,b). Consistently, collagen IV staining also indicated a suppression of fibrosis in the *Nrf2-*KO mice (Fig. 2c).

**Figure 3:**
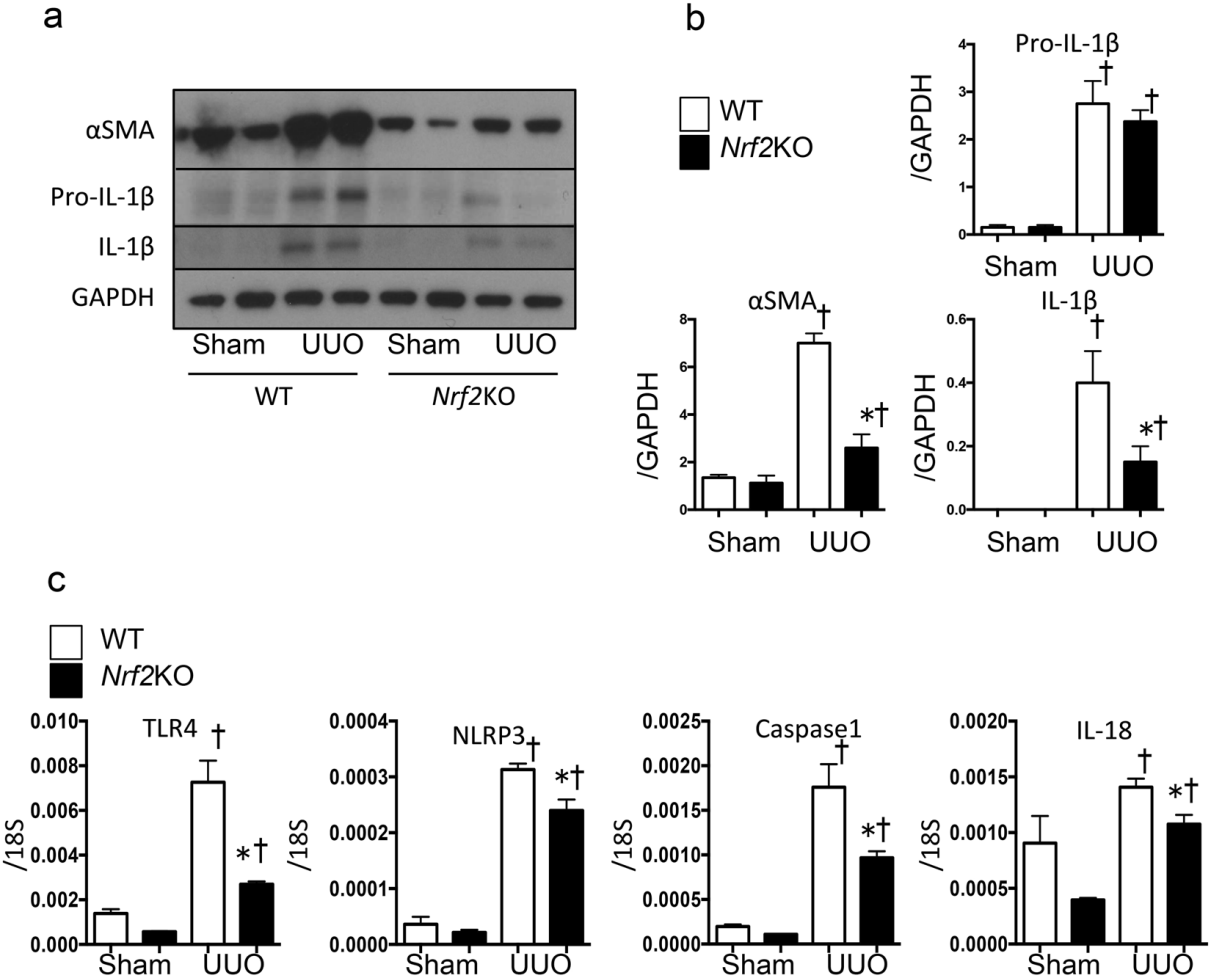
Inflammasome activation in wild-type (WT) and *nuclear factor erythroid 2-related factor 2*-knockout (*Nrf2*-KO) mice after unilateral ureter obstruction. These data were obtained from whole kidney tissues from 6 or more mice in each group. (**a**) and (**b**) Representative western blot data. The following statistical analysis data are shown: *p < 0.05 relative to WT-UUO. ^†^p < 0.05 relative to Sham. (**c**) Expression levels of inflammasome-related genes (*Toll-like receptor 4* [*TLR4*], *nucleotide-binding domain, leucine-rich repeat containing protein* [*NLRP3*], *caspase-1*, and *interleukin-18* [*IL-18*]) were assessed with quantitative reverse transcription PCR (qRT-PCR). The mRNA expression level of each gene was normalized to the expression of 18 S. *p < 0.05 relative to WT-UUO. ^†^p < 0.05 relative to Sham.

When the mRNA levels of two fibrosis-related genes, *connective tissue growth factor* (*CTGF*) and *alpha smooth muscle actin* (*α-SMA*), were evaluated on Day 14 after UUO, the genes showed significantly elevated expression after UUO in the WT kidneys, whereas the gene induction was significantly suppressed in the *Nrf2-*KO-UUO mice compared with the WT mice (Fig. 2d). We also evaluated the amount of the fibrosis protein α-SMA and the inflammation protein IL-1β by western blot analysis (Fig. 3a,b). Although the α-SMA and IL-1β proteins increased markedly upon UUO in the WT mice, the increase in protein levels was less evident in the *Nrf2-*KO-UUO mice compared with the WT-UUO mice (Fig. 3a,b).

Inflammasome activation has been shown to be involved in the progression of inflammation and fibrosis induced by UUO^10^. This finding led us to examine the mRNA levels of inflammation-related genes, including *Toll-like receptor 4* (*TLR4*), *NLR family pyrin domain-containing 3* (*NLRP3*), *caspase 1*, and *IL-18*. Similar to the previous sets of genes, the UUO-induced upregulation of *TLR4* and *NLRP3* was attenuated in the *Nrf2-*KO mice (Fig. 3c). These results indicate that the loss of *Nrf2* suppresses inflammation in UUO.

### M1 macrophages infiltrate the kidney after UUO

We focused on the role of Nrf2 in macrophages in kidney disease because the number of infiltrated macrophages decreased in the *Nrf2*-KO mice compared with the WT mice by Day 14 after UUO (Supplementary Fig. S1). These data encouraged us to focus on the characteristics of macrophages. Two macrophage subtypes, M1 and M2, are known to reciprocally control inflammatory responses. To understand the role of Nrf2 in macrophage function, we sought to characterize the behaviors of these macrophage subtypes during UUO-induced kidney fibrosis using WT mice.

We first harvested bone marrow cells from *green fluorescent protein* (*GFP*) transgenic mice (*GFP*TG) to transplant into the tail veins of the WT mice. We then sorted macrophages from the whole kidney based on the cell-surface expression of the macrophage markers CD11b and F4/80. As shown in Supplementary Figure 2a, the CD11b^+^-F4/80^low^ and CD11b^+^-F4/80^high^ cell populations were GFP-positive, which indicated that these cells were bone marrow–derived macrophages. We noted that the two cell populations with CD11b^+^-F4/80^low^ and CD11b^+^-F4/80^high^ increased by Day 3 of UUO, as previously reported^25^. The CD11b^+^-F4/80^high^ cell group increased further by UUO-Day 7. We sorted CD11b^+^-F4/80^low^ and CD11b^+^-F4/80^high^ with fluorescence-activated cell sorting (FACS) and examined the mRNA expression of the M1 marker, iNOS, and the M2 marker, CD206, with quantitative reverse transcription PCR (qRT-PCR). The higher iNOS mRNA expression in CD11b^+^-F4/80^low^ indicated that this population represented M1 macrophages, whereas the higher CD206 expression in CD11b^+^-F4/80^high^ suggested that these were M2 macrophages (Supplementary Fig. 2b). Based on these data, we concluded that M1 macrophages infiltrate the kidney tissue at an early stage (Day 3) and that M2 macrophages increase after Day 7 upon UUO.

### Nrf2-dependent inflammasome activation contributes to maintaining M1 macrophages in UUO-induced kidney inflammation

**Figure 4:**
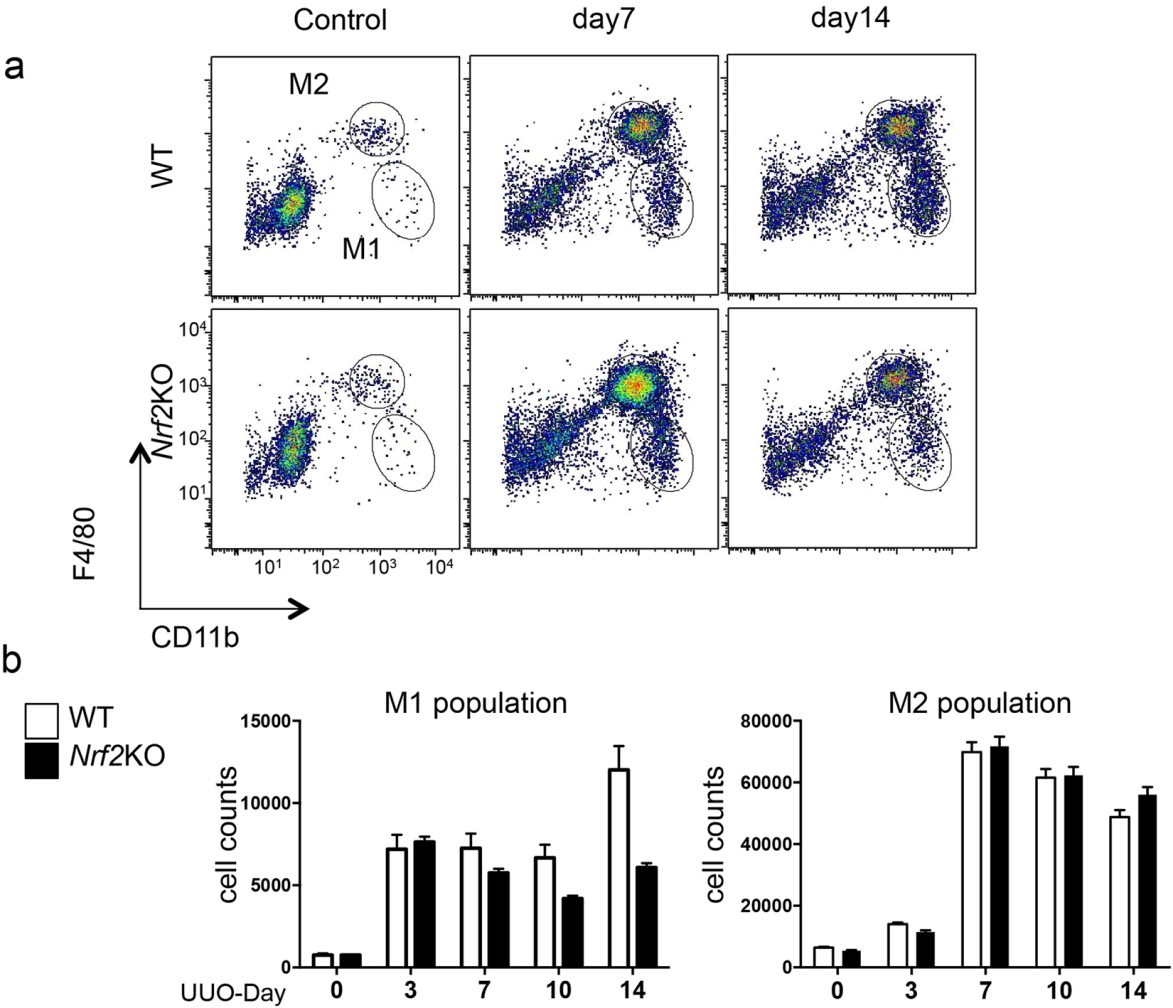
Macrophage polarization in wild type-unilateral ureter obstruction (WT-UUO) mice and *nuclear factor erythroid 2-related factor 2*-knockout unilateral ureter obstruction (*Nrf2*-KO-UUO) mice. These data were obtained from whole kidney tissues from 4 mice in each group. (**a**) Representative flow cytometry data are shown. (**b**) The absolute cell numbers of the M1 and M2 populations after live cell gating. *p < 0.05 relative to WT-UUO on the same day after UUO.

We then examined the time course of M1 and M2 macrophage infiltration in the WT and *Nrf2-*KO mice. We did not detect a substantial difference between the genotypes in either the M1 or M2 macrophage numbers until Day 7 after UUO. However, the *Nrf2-*KO kidneys showed substantially lower numbers of M1 macrophages at Day 14 after UUO compared to the WT kidneys (Fig. 4a,b). When we sorted M1 macrophages from the WT and *Nrf2-*KO mice on Day 7 and investigated the expression of the inflammasome-related genes, the mRNA levels of *IL-1β* and *caspase 1* in M1 macrophages were significantly suppressed in the *Nrf2-*KO samples compared with the WT samples (Fig. 4c), thus demonstrating that *Nrf2* is necessary for the induction of inflammasome-related genes within M1 macrophages.

**Figure 5:**
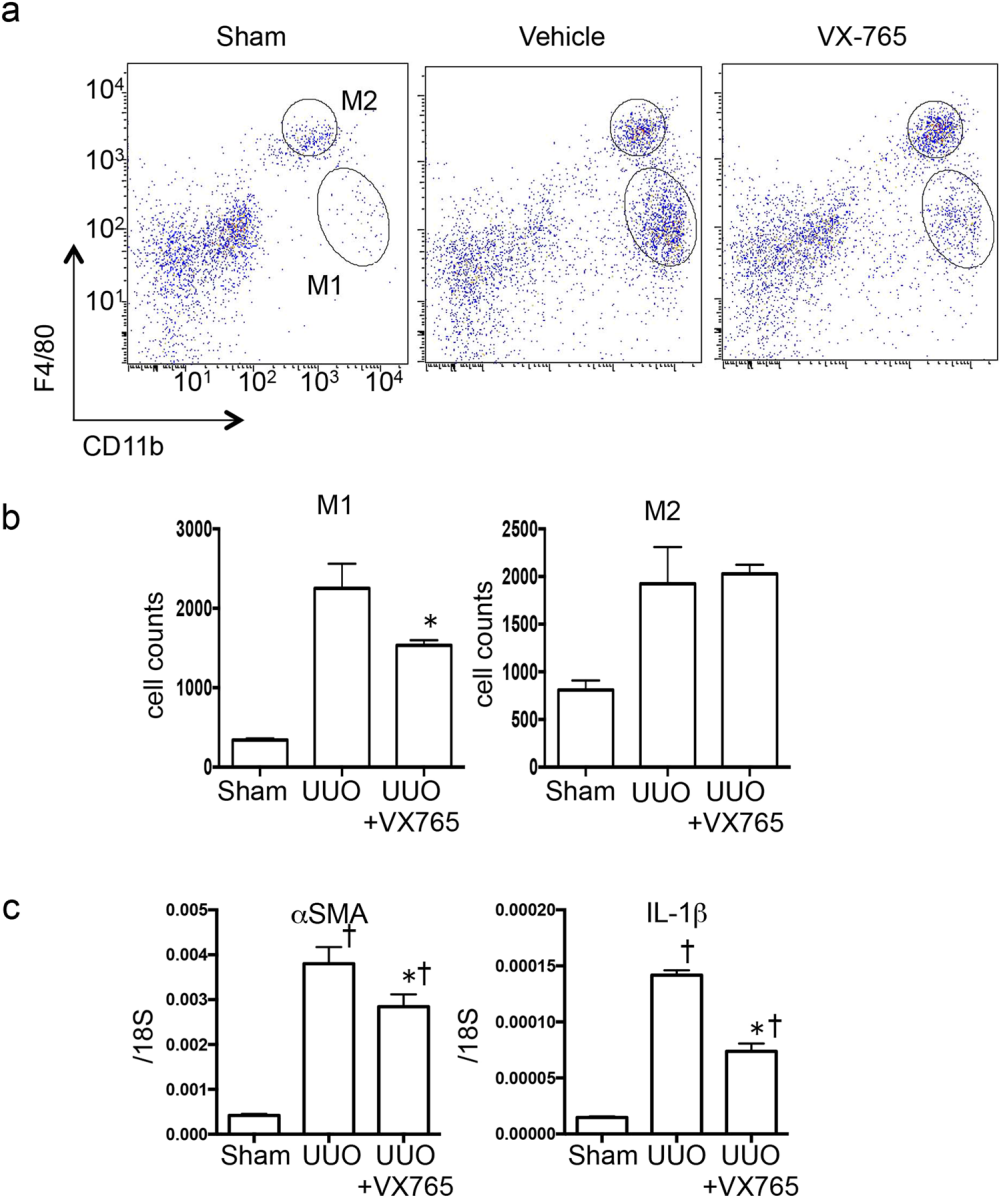
Infiltration of M1 was inhibited with a caspase 1-specific inhibitor, VX765, administered in unilateral ureter obstruction. These data were obtained from whole kidney tissues from 3–4 mice in each group. (**a**) Representative flow cytometry data are shown. (**b**) The absolute cell numbers of the M1 and M2 populations after the live cell gating. *p < 0.05 relative to vehicle group. ^†^p < 0.05 relative to Sham. (**c**) RNA was isolated from whole kidney tissues from each group. *Alpha smooth muscle actin* (α-SMA) and *interleukin-1β* (IL-1β) were assessed with quantitative reverse transcription PCR (qRT-PCR). The mRNA expression level of each gene was normalized to the expression of 18S. *p < 0.05 relative to vehicle group. ^†^p < 0.05 relative to Sham.

To determine the roles of inflammasome activation in M1 macrophage infiltration, we administered the caspase 1-specific inhibitor VX765 orally during the later phase of UUO (Days 7–14) and investigated the maintenance of the M1 macrophage population. The VX765 treatment in the later stage was sufficient to decrease the M1 macrophage population (Fig. 5a,b). The mRNA expression of *IL-1β* and *α-SMA* was also suppressed significantly by VX765 treatment (Fig. 5c). These results indicate that inflammasome activation is important for maintaining the M1 macrophages after their initial infiltration and that *Nrf2* may contribute to this process.

**Figure 6:**
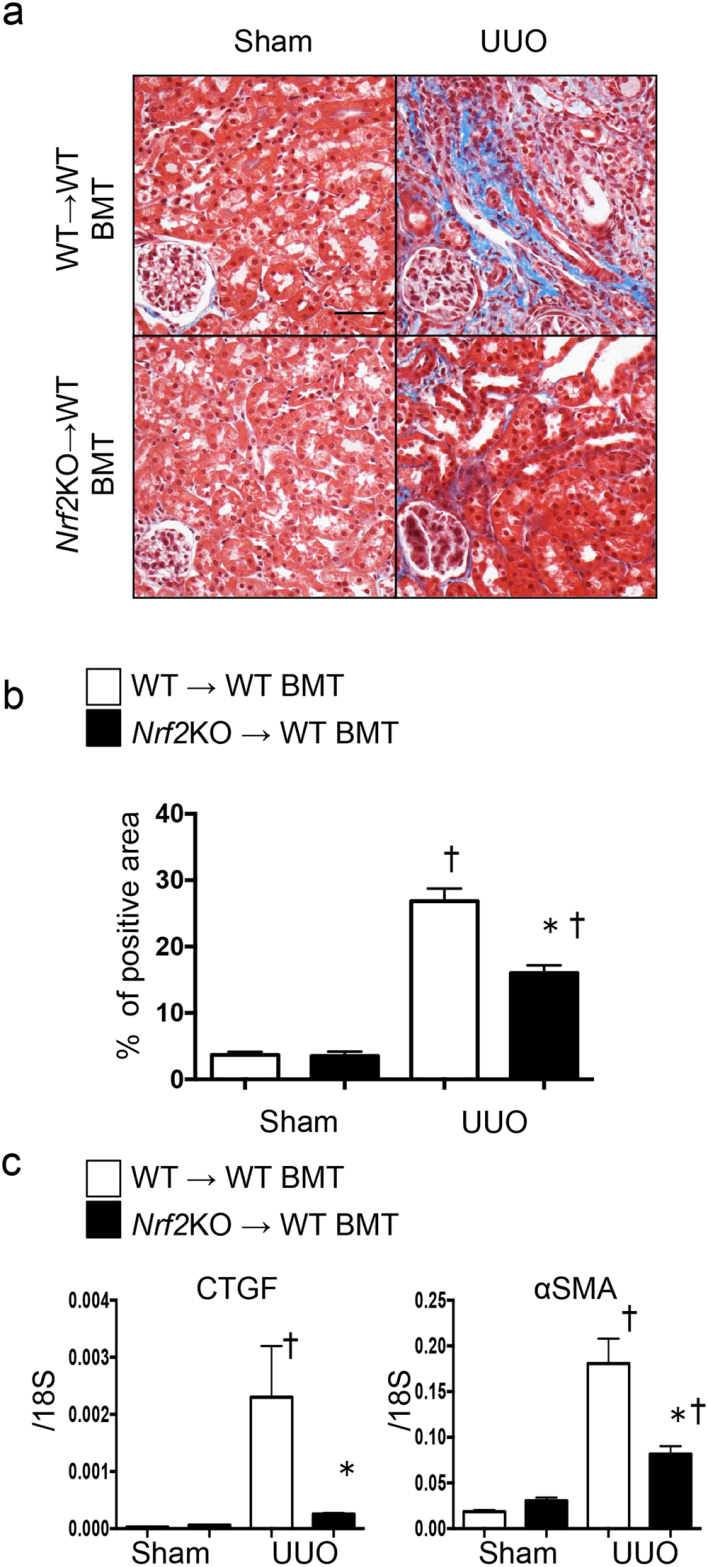
Fibrosis in unilateral ureter obstruction (UUO) after bone marrow transplantation (BMT). BMT was performed to determine the role of *Nrf2* in macrophages. These data were obtained from whole kidney tissues from 6 or more mice in each group. (**a**) Fibrosis was assessed with Masson’s trichrome staining. Representative images are shown. (**b**) The tubulointerstitial fibrosis area (%) was evaluated as the blue-stain-positive area in Masson’s trichrome staining. Scale bar = 50 μm. *p < 0.05 relative to BMT (wild-type [WT] → WT)-UUO. ^†^p < 0.05 relative to Sham. (**c**) *Connective tissue growth factor* (CTGF) and *alpha smooth muscle actin* (α-SMA) were assessed by quantitative reverse transcription PCR (qRT-PCR). *p < 0.05 relative to BMT (WT → WT)-UUO. ^†^p < 0.05 relative to Sham.

**Figure 7:**
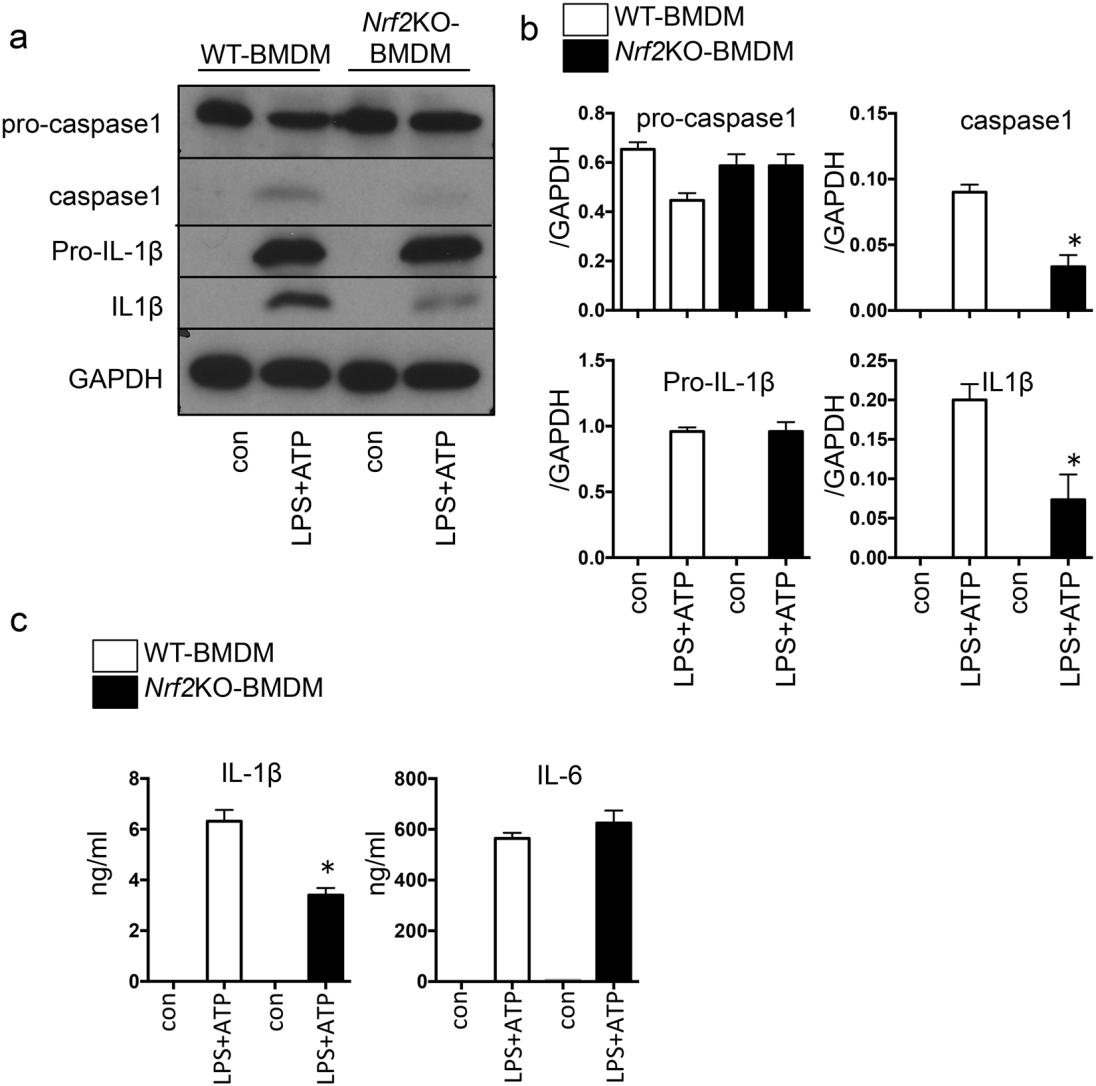
The role of *nuclear factor erythroid 2-related factor 2* (*Nrf2*) in bone marrow–derived macrophages (BMDMs). The *in vitro* assays using bone marrow–derived macrophages (BMDMs) were performed at least 3 times. Each experiment was performed in duplicate. (**a**) and (**b**) Representative western blot data from cell lysates. The following statistical analysis data are shown: (**c**) The levels of interleukin-1β (IL-1β) and IL-6 were detected with enzyme-linked immunosorbent assay (ELISA) in supernatant. *p < 0.05 relative to lipopolysaccharide (LPS) and adenosine triphosphate (ATP) stimulation for wild-type (WT)-BMDMs.

Next, we tested whether the role of *Nrf2* in kidney inflammation is cell autonomous within macrophages. We transplanted cells from either *Nrf2-*KO or *Asc-*KO bone marrow into WT bone marrow. We then evaluated kidney fibrosis on Day 14 after UUO with Masson’s trichrome staining. The UUO-induced kidney fibrosis was significantly suppressed in the kidneys of the WT animals that received *Nrf2-*KO bone marrow cells (Fig. 6a,b). The mRNA levels of the fibrosis genes *α-SMA* and *CTG*F were significantly reduced in the animals with transplanted *Nrf2-*KO bone marrow cells compared with those with transplanted WT cells (Fig. 6c). We obtained similar results upon the transplantation of *Asc-*KO bone marrow cells (Supplementary Fig. S3a,b), which indicated that inflammasome activation drives the progression of fibrosis. To investigate the direct role of *Nrf2* in the activation of NLRP3 inflammasomes, we isolated bone marrow–derived macrophages (BMDMs) from the WT and *Nrf2-*KO mice. We then treated the BMDMs with lipopolysaccharide-adenosine triphosphate (LPS-ATP) and measured the activation of NLRP3 inflammasomes by monitoring the protein levels of the cleavage of caspase 1 and IL-1β in the BMDMs via western blot analysis and by monitoring the secretion of IL-1β and IL-6 into the supernatant with enzyme-linked immunosorbent assay (ELISA). We found decreased caspase 1 cleavage and a reduced secretion of IL-1β from the *Nrf2-*KO BMDMs compared with the WT cells (Fig. 7a–c). However, the secretion of IL-6, which is expressed by the Nfκb pathway, did not differ between the genotypes, which suggests that Nrf2 plays a specific role in inflammasome activation. These data demonstrate that Nrf2 is directly involved in the activation of NLRP3 inflammasomes within macrophages.

## Discussion

In this study, we elucidated two main points concerning the role of *Nrf2* in the progression of kidney disease. First, Nrf2 activation of macrophages is essential for inflammasome activation. In an *Nrf2*-deficient murine kidney disease model, the prolongation of inflammation was suppressed, and fibrosis was ameliorated. Nrf2-related NLRP3 inflammasome activation was important for maintaining the M1 population in this disease model and, as a result, seems to contribute to the process by which organ inflammation becomes chronic inflammation.

The importance of inflammasome activation in the progression of kidney disease, including UUO, has already been reported^26^. Many studies have examined the role of inflammasome activation and an upstream pathway in the progression of kidney disease. We have reported that aldosterone caused inflammasome activation via macrophage mitochondrial dysfunction^27^. We also reported that the mitochondrial redox response could induce inflammasome activation in nephrotic syndrome mice^28^. However, no reports have suggested how inflammasome activation contributes to the progression of kidney disease in these disease states. Our data also show that M1 increases on UUO-Day 3 and that M2 was already prominent on Day 7. Fujiu *et al*. performed a similar study and showed that M2 becomes dominant at an early stage^25^. It is particularly interesting that a continued observation of the M1 population at Day 14 showed almost no changes in the WT mice but a clear decrease in the *Nrf2-*KO and *Asc-*KO mice. In the *Nrf2*-deficient cells, the NLRP3 inflammasome might be activated after UUO *in vivo* or after stimulation *in vitro*. This finding suggests that there is both Nrf2-related and Nrf2-unrelated NLRP3 inflammasome activation. Nrf2-dependent inflammasome activation may be involved in maintaining the M1 population. The mechanism is unclear, but it has been reported that high-mobility group protein box 1 (HMGB1) is involved in inducing the M1 population^29, 30^, and it is possible that HMGB1 and extracellular histones act as damage-associated molecular pattern (DAMP) molecules^31^. Although the association of cytokines such as IL-1β also plays an important role in inflammasome activation, it has become clear that DAMPs such as HMGB1 are extremely important in enhancing the progression of inflammation^32^. Because HMGB1 or extracellular histone activates TLR4 signaling^33^, this action may contribute to the induction of macrophages to M1^34^. This kind of *in vivo* vicious cycle may be a factor underlying why pathologies become chronic, and it is an important discovery because the suppression of inflammasome activation may become a viable therapeutic target.

The results of this study suggest that Nrf2-dependent inflammasome activation plays an important role in the pathogenesis of kidney disease and that it may contribute to the chronicity of the disease. A particularly interesting result is that maintenance of the M1 population associated with inflammasome activation later causes fibrosis. However Nrf2 activators have the potential to be effective new drugs in various fields^35, 36^. The results of this study should not discourage therapeutic intervention with Nrf2-activating drugs. The mechanism of action of the Nrf2 activators has not been clarified in previous studies, and the possibility of direct involvement—that is, without the involvement of transcriptional activity—has also been suggested^7^.

It has been shown that the expression of many anti-oxidant and anti-inflammatory gene clusters is controlled by Nrf2 activation. It can be imagined that an upregulation of such gene cluster expression would have an organ-protective effect; Nrf2 activators are thus garnering attention as potential new drugs in various fields^35, 36^. In kidney diseases, several reports have shown that the activation of Nrf2 ameliorates kidney dysfunction by inhibiting inflammasome activation^5, 37^. The redox status that is produced by mitochondrial dysfunction and NAD(P)H oxidase activation could induce NLRP3 inflammasome activation. However, in this study, organ fibrosis was actually suppressed in *Nrf2*-deficient mice. One reason for this could be that the expression of anti-oxidant (e.g., *SOD2*, *NQO1*) clusters is already suppressed in this model. *NQO*1 and *S100a9* are *Nrf2*-dependent genes because the expression of these genes disappeared in the *Nrf2*-KO mice before and after UUO (Fig. S5). There was no statistically significant difference between the WT-UUO and *Nrf2-*KO-UUO mice (Supplementary Figs S4 and S5). For *S100a9*, another Nrf2-related gene, the expression increased in related with Nrf2 protein expression (Supplementary Figs S4 and S5), and it was suggested that gene expression associated with Nrf2 activation may vary widely across different diseases.

Another possibility is that there might be several types of mechanism of NLRP3 inflammasome activation and that some – but not all –are dependent on *Nrf2*. CD36 could be a key molecule for understanding the relationship between Nrf2 and inflammasome activation. Nrf2 regulates the expression of CD36 in macrophages^38^. In addition, Sheedy *et al*. reported that CD36 has a pivotal role in NLRP3 inflammasome activation^39^. In kidney disease, a CD36 antagonist has been shown to prevent disease progression^40^.

Based on the results of this study, Nrf2-dependent inflammasome activation plays an important role in the progression of kidney disease. This activation had a particularly strong effect on the persistence of M1 macrophage infiltration. These novel results contribute to elucidating the pathology of chronic kidney disease, especially prolonged inflammation.

## Materials and Methods

All methods were performed in accordance with the relevant guidelines and regulations.

### Animals

The experimental protocols (no. 15–061, no. 15–077 and no. 15–112) were approved by the Animal Research Committee of Kawasaki Medical School, which is based on the National Institutes of Health Guide for the Care and Use of Laboratory Animals (NIH Publication No. 80–23, revised 1996). Eight-week-old male mice weighing 20 to 30 g at the beginning of the study were designated wild-type (WT). *GFP*-TG mice were kindly provided by Y. Sunada (Neurology Department, Kawasaki Medical University, Okayama, Japan)^41^. *Asc* homozygous knockout (*Asc-*KO) mice were kindly provided by M. Takahashi (Jichi Medical University, Shimotsuke, Japan)^8^. *Nrf2* mutant (*Nrf2-*KO) mice were purchased from RIKEN (Ibaraki, Japan)^24^. All mice underwent unilateral ureteral obstruction (UUO) or a sham operation (sham). All mice were from the C57B/6 J background.

### Bone marrow transplantation

Bone marrow transplantation (BMT) was performed according to a standard protocol described previously^27, 41^. Using this protocol, four types of chimeric mice were obtained as follows: BMT (WT → WT), BMT (*GFP* → WT), BMT (*Asc-*KO → WT), and BMT (*Nrf2-*KO → WT) mice.

### Cell culture

Bone marrow–derived macrophages (BMDMs) were used for *in vitro* assays. Bone marrow cultures were prepared using Macrophage colony-stimulating factor (MCSF)-containing media^42^. To activate the NLRP3 inflammasome, BMDMs were primed for 3 hours with ultrapure LPS (InvivoGen, San Diego, CA, USA), followed by stimulation with ATP (5 mM) 30 min before the cell lysates and supernatants were harvested. IL-1β and IL-6 were detected using ELISA (R&D Systems, Minneapolis, MN, USA).

### Western immunoblotting

Kidney and cell lysates were extracted with extraction buffer or sample buffer as described previously^43^. Protein samples were subjected to immunoblotting analysis with antibodies against α-SMA (100M4795; Sigma-Aldrich, St Louis, MO, USA), ASC (sc-22514-R; Santa Cruz Biotechnology, Dallas, TX, USA), caspase-1 (sc-514; Santa Cruz Biotechnology), F4/80 (MCA497GA; AbD Serotec, Raleigh, NC, USA), IL-1β (ab9722; Abcam, Cambridge, MA, USA), Nrf2 (sc-13032; Santa Cruz Biotechnology) and GAPDH (sc-25778; Santa Cruz Biotechnology). Signals were detected using an enhanced chemiluminescence system (GE Healthcare Japan, Tokyo, Japan). Each western blot was performed on a pooled sample of 4–6 mice from each group.

### RNA extraction and quantitative reverse transcription PCR

The protocols for extracting RNA and making cDNA were described in a previous report^44^. The primers and probes for the TaqMan analysis were designed using sequence information from GenBank (National Institutes of Health, Bethesda, MD, USA)^45^ and Primer3 online software (http://frodo.wi.mit.edu/primer3/; accessed July 1, 2015). The primer and probe sequences are listed in the Supplementary Table in the supplemental information. TaKaRa Premix Ex Taq (Takara Bio, Inc., Otsu, Japan), with a final reaction volume of 20 μl, was used for the TaqMan probe-based quantitative reverse transcription PCR (qRT-PCR) reaction, which was performed on an Applied Biosystems 7500 Fast Real-Time PCR System (Applied Biosystems; Thermo Fisher Scientific, Inc.).

The level of mRNA expression in each sample was quantified using the absolute quantification standard curve method^46^. The plasmid cDNA of each gene was used to prepare the absolute standards. The concentration was measured using the A260, which was converted to the number of copies using the molecular weight of the DNA. Each mRNA expression level was normalized to that of the housekeeping 18 S ribosomal RNA gene. The levels of expression in each sample were normalized to that of 18S RNA.

### Flow cytometric analysis

Kidney cells were prepared according to a protocol described previously^25^. Kidney tissue was immediately homogenized using gentleMACS™ (Miltenyi Biotec, Tokyo, Japan) with collagenase-containing extraction buffer. The cells were dissolved in FACS buffer to perform FACS analysis using a FACS CantoII^TM^ (BD Bioscience, Tokyo, Japan) and FlowJo software (Tree Star, Ashland, OR, USA). An allophycocyanin (APC)-labeled anti-mouse F4/80 antibody (B202565; BioLegend, San Diego, CA, USA) and a phycoerythrin (PE)-labeled anti-mouse/human CD11b antibody (B194776; BioLegend) were used. Cell sorting was performed with FACSAria^TM^ III (BD Bioscience) for RNA extraction using an RNeasy Kit (Qiagen, Germantown, MD, USA).

### Histopathological examination

Half of the kidneys were fixed in 4% paraformaldehyde and embedded in paraffin for histological analysis. Masson trichrome–stained specimens were observed under an inverted microscope (BZ-9000, Keyence, Osaka, Japan). The percentage of blue-stained scarred areas was quantified using a color image analyzer (WinROOF; Mitani, Fukui, Japan)^47^.

### Immunohistochemical staining

For immunohistochemical staining, serial cryostat sections (4 µm thick) of paraffin-embedded specimens were rehydrated in phosphate buffered saline (PBS) and subjected to antigen retrieval in a microwave. Antibodies against collagen IV (ab6586; Abcam, Cambridge, MA, USA) and F4/80 (MCA497GA; AbD Serotec) were used, and detection was carried out using the Histofine Simple Stain^TM^ MAX PO (MULTI) kit (Nichirei, Tokyo, Japan) and 3,3-diaminobenzidine (Sigma-Aldrich).

### Statistical analyses

All values are expressed as the means ± standard error of the mean (SEM). Statistical analyses were calculated using GraphPad Prism6 software (GraphPad Software, La Jolla, CA, USA). Comparison between multiple groups was performed by using one-way ANOVA. P values less than 0.05 were considered statistically significant.

## Electronic supplementary material

Supplementary info
