# Supplementary material for: Infiltration of M1, but not M2, macrophages is impaired after unilateral ureter obstruction in Nrf2-deficient mice
Source: Sci Rep. 2017 Aug 18;7:8801. doi: 10.1038/s41598-017-08054-2 (PMC5562821; doi:10.1038/s41598-017-08054-2)

Supplementary information

Title: Infiltration of M1, but not M2, macrophages is impaired after unilateral ureter obstruction in Nrf2-deficient mice.

Yuji Sogawa[1,2], Hajime Nagasu[1,2], Shigeki Iwase[3], Chieko Ihoriya[1], Seiji Itano[1], Atsushi Uchida[1], Kengo Kidokoro[1], Shun'ichiro Taniguchi[4], Masafumi Takahashi[5], Minoru Satoh[1], Tamaki Sasaki[1], Takafumi Suzuki[6], Masayuki Yamamoto[6], Tiffany Horng[7], Naoki Kashihara[1]

Supplementary legends

Figure S1: F4/80 staining in WT and *Nrf2*KO.

These data were obtained from whole kidney tissue from 10 or more mice in each group. F4/80 staining was performed to detect infiltration of macrophages. a) Representative picture were shown from WT and Nrif2KO kidney after unilateral ureter obstruction (UUO). And F4/80 positive area were cuculated to assess the infiltration of macrophages in kidney after UUO. * $p<0.05$ relative to WT-UUO b) Expression of F4/80 was assessed with quantitative RT-PCR. * $p<0.05$ relative to WT-UUO. † $p<0.05$ relative to Sham.

Figure S2: Population of macrophages in UUO kidney. These data were obtained from whole kidney tissue from 3 mice in each group.

a) Representable FACS data were shown. Upper panels are GFP positive cells and lower panels are GFP negative cells. b) Expression of CD206 and iNOS mRNA levels were assessed by qRT-PCR. Expression of each mRNA level was normalized to 18S expression. Each population was sorted from GFP positive cells. * $p<0.05$ relative to $CD11b^{+}$-$F4/80^{low}$.

Figure S3: Renal fibrosis in WT→WT BMT UUO and *ASC*KO→WT BMT UUO.

These data were obtained from whole kidney tissue from 3 mice in each group. a) Fibrosis was assessed by Masson trichrome staining in kidney 14 days after UUO. Representative picture was shown. b) Fibrosis-associated genes (CTGF and α SMA) were assessed by qRT-PCR. * $p<0.05$ relative to WT→WT BMT UUO.

Figure S4: Trend of Nrf2-dependent gene expression in WT-UUO kidney.

These data were obtained from whole kidney tissue from 3 mice in each group. Expression of SOD2, NQO1 and S100a9 mRNA levels were assessed by qRT-PCR. Expression of each mRNA levels was normalized to 18S expression.

Figure S5: Nrf2-dependent gene expression in WT-UUO and Nrf2KO-UUO kidney.

These data were obtained from whole kidney tissue from 6 mice in each group. Expression of SOD2, NQO1 and S100a9 mRNA levels were assessed by qRT-PCR. Expression of each

mRNA levels was normalized to 18S expression. * $p<0.05$ relative to WT-UUO. † $p<0.05$ relative to Sham.

Supplementary Table.

Gene	Accession number	Primer and TaqMan probe sequences (5'-3')
αSMA	NM_007392	Forward primer: CAGGCATTGCTGACAGGAT
		Reverse primer: GTTCTGGAGGGGCAATGAT
		TaqMan probe: FAM- CTCGCACCCAGCACCATGAAGA -TAMRA
Caspase1	NM_009807	Forward primer: ACCCTCAAGTTTTGCCCTTT
		Reverse primer: CCCTCGGAGAAAGATGTTGA
		TaqMan probe: FAM- CCACTCGTACACGTCTTGCCCTCA -TAMRA
CD206	NM_008625	Forward primer: AACAAGAATGGTGGGCAGTC
		Reverse primer: TTTGCAAAGTTGGGTTCTCC
		TaqMan probe: FAM- TCATTTGGATGGATGGGAGCAAA -TAMRA
CTGF	NM_010217	Forward primer: TACCGTGGGAGGAACTATCC
		Reverse primer: CTCACCTCAGTGTGCGTTCT
		TaqMan probe: FAM- CAGTTGTTCATTAGCGCACAGTGCC -TAMRA
F4/80	NM_010130	Forward primer: CCTGGCTTTGCATCTAGCA
		Reverse primer: AGGAGCCTGGTACATTGGTG
		TaqMan probe: FAM- TTGATGAGTGCACCCAAGATCCA -TAMRA
GCLM	NM_008129	Forward primer: CAATGACCCGAAAGAACTGC
		Reverse primer: ATTCCCCTGCTCTTCACGAT
		TaqMan probe: FAM- TCCCTGACATTGAAGCCCAGGATTG -TAMRA
HO-1	NM_010442	Forward primer: TGCTCGAATGAACACTCTGG
		Reverse primer: AAGGCGGTCTTAGCCTCTTC
		TaqMan probe: FAM- CCTGAGGTCAAGCACAGGGTGACA -TAMRA
IL-18	NM_008360	Forward primer: AGACAGCCTGTGTTCGAGGA
		Reverse primer: AGAGGGTCACAGCCAGTCC
		TaqMan probe:FAM- CAAAGTGCCAGTGAACCCCAGACCA -TAMRA
IL-1β	NM_008361	Forward primer: AGGGCTGCTTCCAAACCT
		Reverse primer: TGCCACAGCTTCTCCACA
		TaqMan probe: FAM- CCTGGGCTGTCCTGATGAGAGCA -TAMRA
iNOS	NM_010927	Forward primer: AGACCTCAACAGAGCCCTCA
		Reverse primer: GGCTGGACTTTTCACTCTGC
		TaqMan probe: FAM- CCATGAGGCTGAAATCCCAGCA-TAMRA
NLRP3	NM_145827	Forward primer: CCTTGGACCAGGTTCAGTGT
		Reverse primer: AGGCAGCAGTTCACCAGTCT
		TaqMan probe: FAM- TCCAGACACTCATGTTGCCTGTTC -TAMRA
NQO1	NM_008706	Forward primer: TTCTCTGGCCGATTCAGAGT
		Reverse primer: TCCAGACGTTTCTTCCATCC
		TaqMan probe: FAM- TTTACAGCATTGGCCACACTCCACC -TAMRA
SOD2	NM_013671	Forward primer: CCAAAGGAGAGTTGCTGGAG
		Reverse primer: GAACCTTGGACTCCCACAGA
		TaqMan probe: FAM- TCAAGCGTGACTTTGGGTCTTTTGA -TAMRA
TLR4	NM_021297	Forward primer: TTTATTCAGAGCCGTTGGTG
		Reverse primer: TGCCTCAGCAGGGACTTC
		TaqMan probe: FAM- CTGAGCAGCCGCTCTGGCATC -TAMRA
18S rRNA	NR_003278	Forward primer: CCTGCGGCTTAATTTGACTC
		Reverse primer: GACAAATCGCTCCACCAACT
		TaqMan probe: FAM- TCTTTCTCGATTCCGTGGGTGGTG -TAMRA

FAM, 6-carboxyfluorescein; TAMRA, N,N,N',N'-tetramethyl-6-carboxyrhodamine derivative.

Supplementary-Figure S1

a

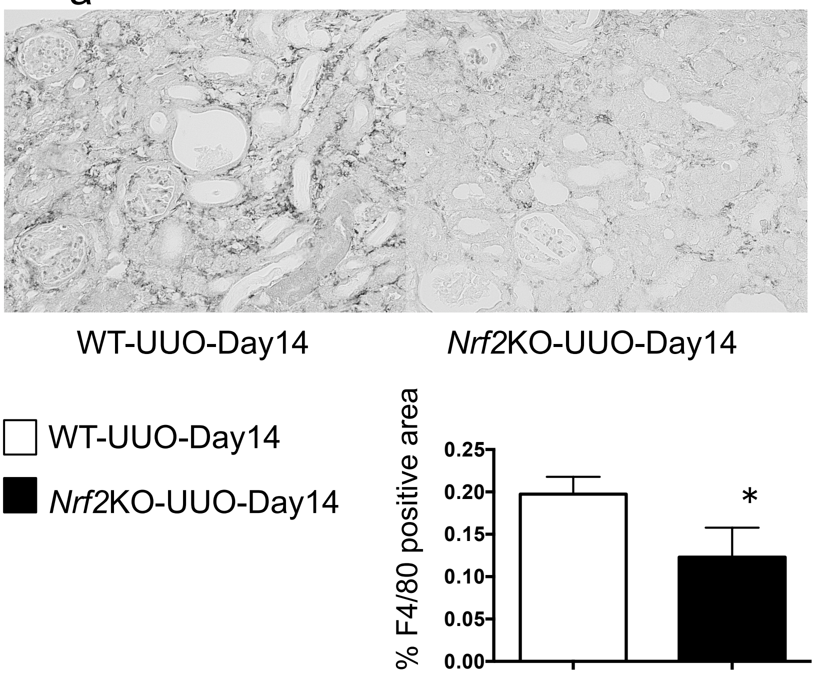

b

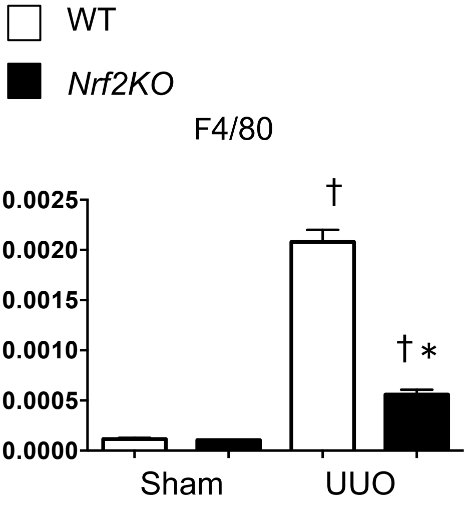

Supplementary-Figure S2

a

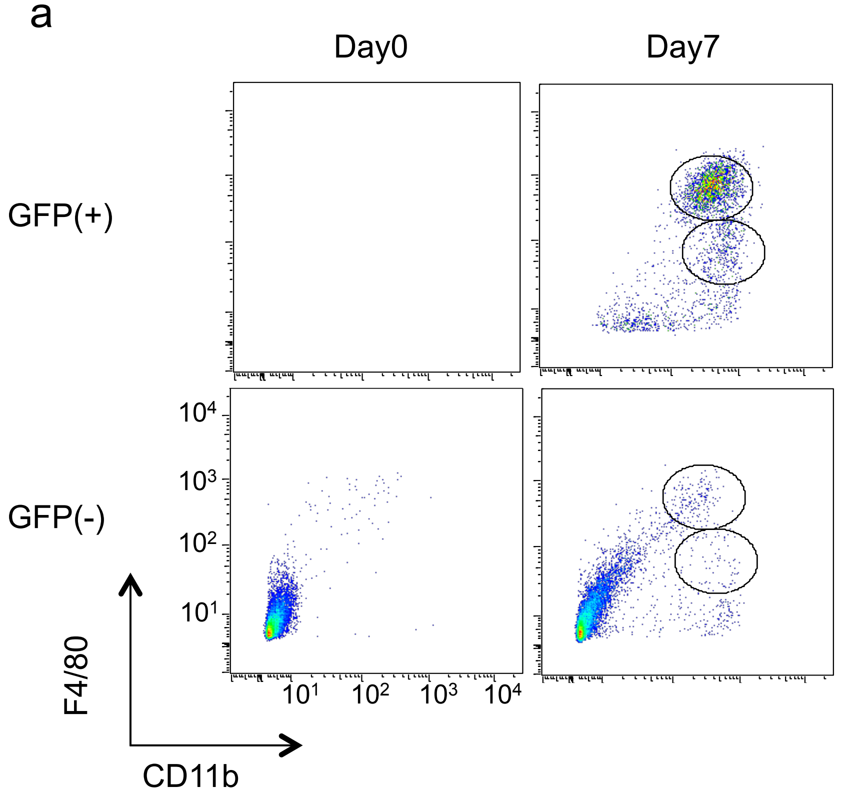

b

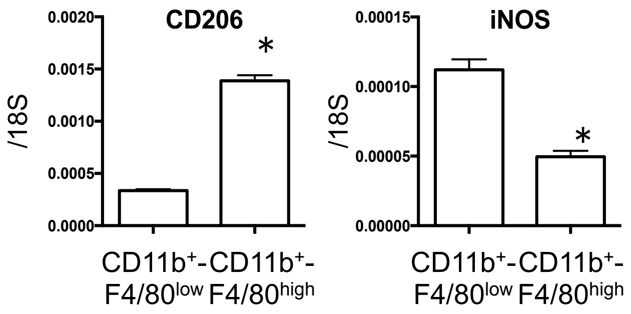

Supplementary-Figure S3

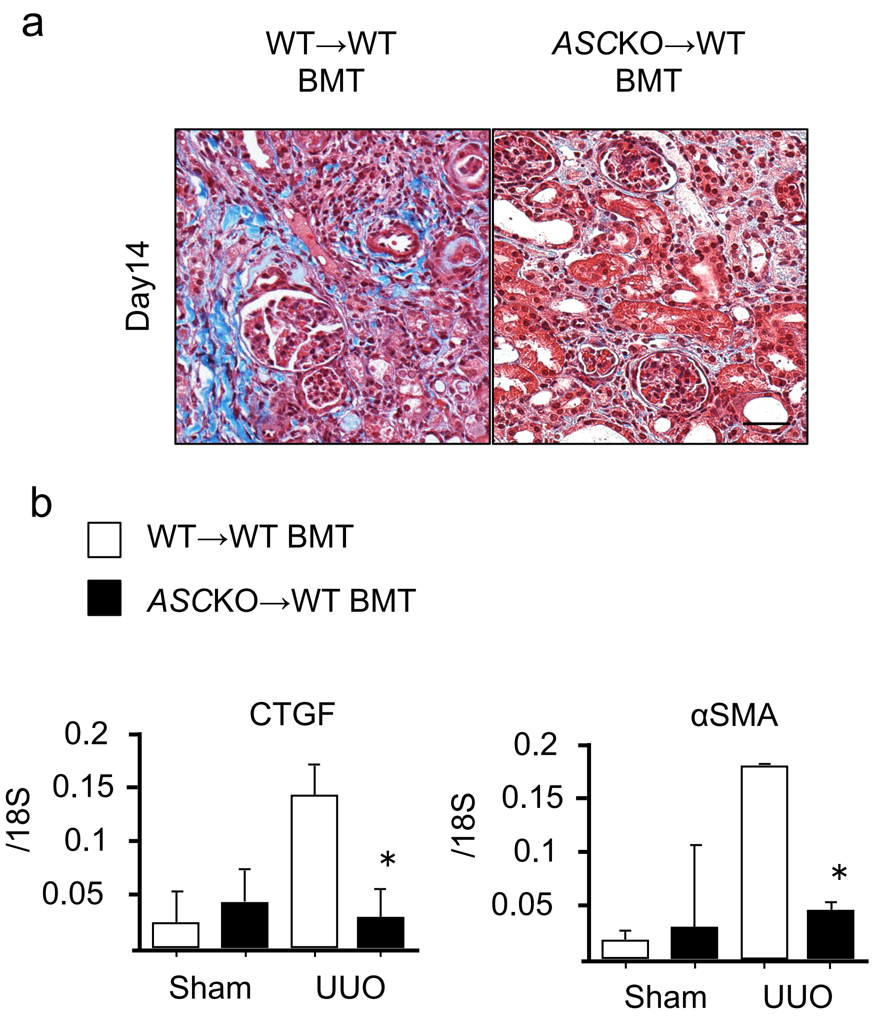

Supplementary-Figure S4

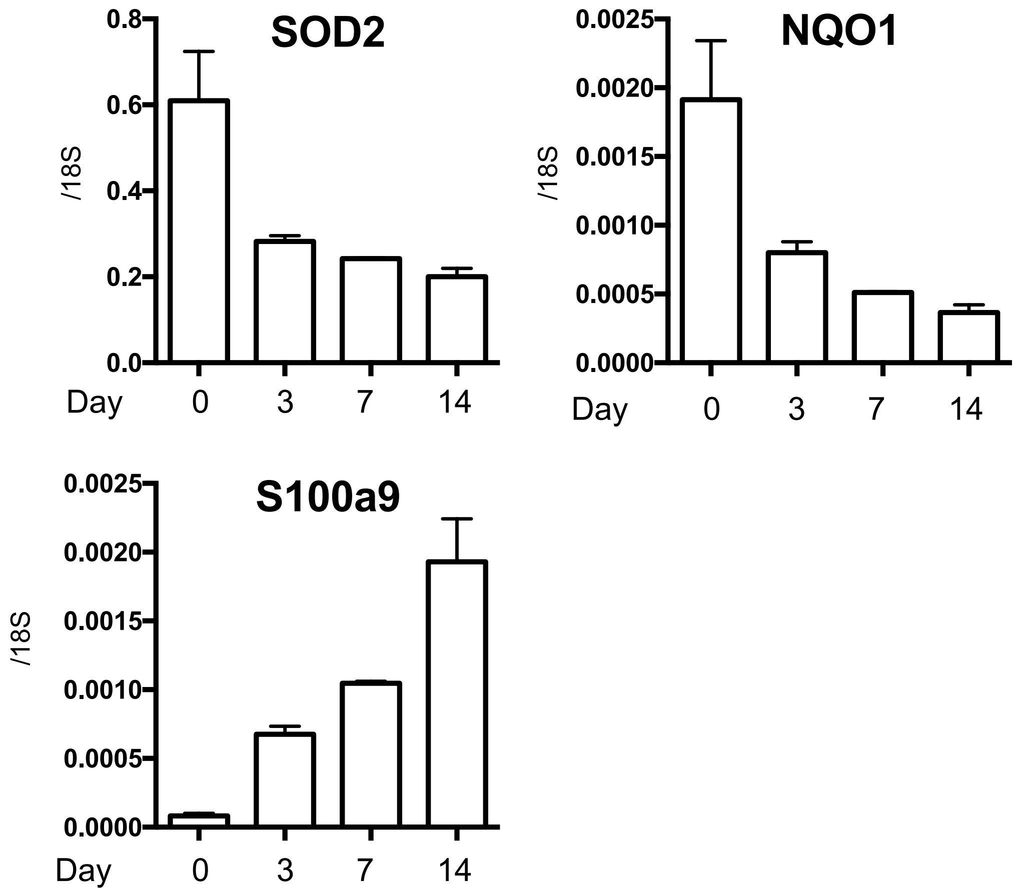

Supplementary-Figure S5

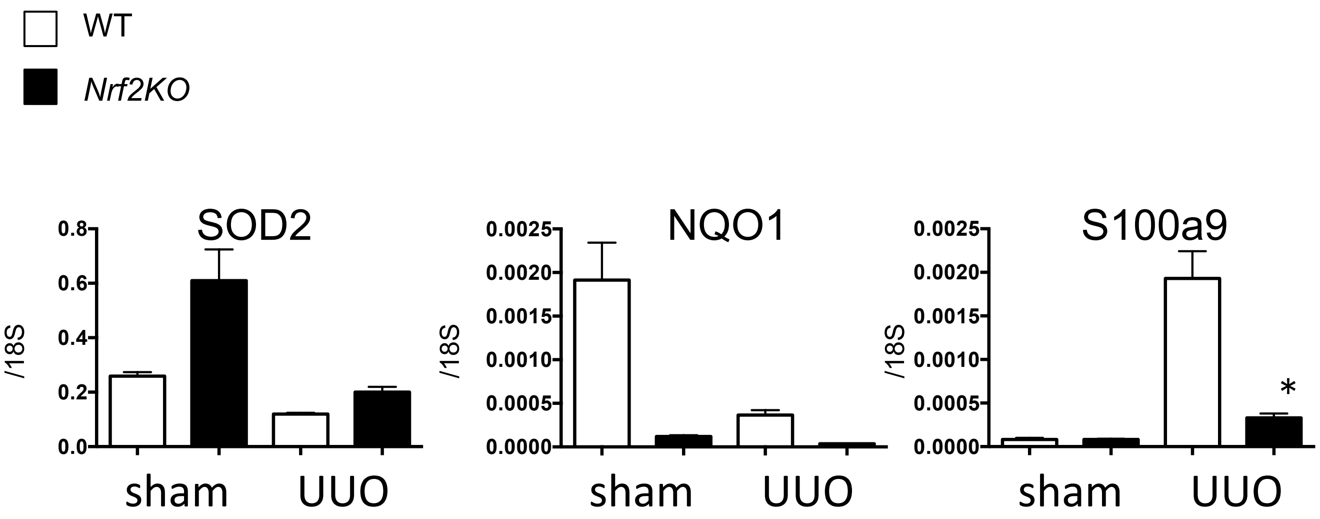

Supplement: Supplementary file 1 — Supplementary info [file 41598_2017_8054_MOESM1_ESM.pdf]
